# Cost-effectiveness of targeted next-generation sequencing (tNGS) for detection of tuberculosis drug resistance in India, South Africa and Georgia: a modeling analysis

**DOI:** 10.1016/j.eclinm.2024.103003

**Published:** 2024-12-24

**Authors:** Suvesh Shrestha, Angelina Addae, Cecily Miller, Nazir Ismail, Alice Zwerling

**Affiliations:** a School of Epidemiology and Public Health, University of Ottawa, Ottawa, Canada; b Global Tuberculosis Program, World Health Organization, Geneva, Switzerland; c Department of Clinical Microbiology and Infectious Diseases, Faculty of Health Sciences, Wits University, Johannesburg, South Africa

**Keywords:** Targeted next-generation sequencing (tNGS), Drug-resistant tuberculosis (DRTB), Cost-effectiveness analysis, Incremental cost-effectiveness ratio (ICER), Disability-adjusted life year (DALY)

## Abstract

**Background:**

Targeted next-generation sequencing (tNGS) is promising alternative to phenotypic drug susceptibility testing (pDST) for detecting drug-resistant tuberculosis (DRTB). This study explored the potential cost-effectiveness of tNGS for the diagnosis of DR-TB across 3 settings: India, South Africa and Georgia.

**Methods:**

To inform WHO guideline development group (GDG) on tNGS we developed a stochastic decision analysis model and assessed cost-effectiveness of tNGS for DST among rifampicin resistance individuals. We also assessed tNGS as initial test for TB drug resistance in bacteriologically confirmed TB. Diagnostic accuracy and cost data were sourced from a systematic review conducted for GDG, covering studies published until September 2022. The primary outcome was incremental cost (2021 US$) per disability-adjusted life year (DALY) averted.

**Findings:**

tNGS when compared with in-country DST, tNGS proved cost-effective in South Africa (ICER: $15,619/DALY averted, WTP: $21,165) but not in Georgia (ICER: $18,375/DALY averted, WTP: $15,069). In India, tNGS dominated in-country DST practice, providing greater health impact at lower cost. When comparing tNGS with universal pDST, tNGS was dominated by pDST in all three countries. In Georgia, using tNGS as initial test for TB drug-resistance compared to Xpert MTB/Rif followed by pDST appeared cost-effective. Scenario with 50% reduction in tNGS test kit costs made tNGS cost-effective across all three countries, while a high Bedaquiline resistance prevalence (30%) led to a worsening cost-effectiveness.

**Interpretation:**

tNGS may be cost-effective in India, South Africa and Georgia when comprehensive DST is not routinely performed. Thus, existing DST practice and healthcare infrastructure should be considered before implementation and scale-up of tNGS.

**Funding:**

Global Tuberculosis Program, 10.13039/100004423World Health Organization (2022/1249364-0).

**Research in context**

**Evidence before this study**

Before undertaking this study, we conducted a systematic review on the economic evaluation of using targeted next-generation sequencing (tNGS) to diagnose drug-resistant tuberculosis (DR-TB). Our search, conducted on Aug 14, 2024, included databases such as PubMed, EMBASE, and SCOPUS, without restrictions on publication year, age group, country, income level, comparator group, HIV status, or other comorbidities. The search revealed no prior cost-effectiveness analyses (CEA) specifically focused on the use of tNGS for the diagnosis of DR-TB.

**Added value of this study**

This study is the first to conduct a cost-effectiveness analysis of tNGS for the detection of DR-TB in low- and middle-income countries (LMICs). It provides essential economic evidence that has informed the World Health Organization (WHO) in making recommendations regarding the adoption of this novel diagnostic tool. This study highlights the potential of tNGS to improve patient outcomes and offers valuable insights into its cost-effectiveness in diverse healthcare settings.

**Implications of all the available evidence**

Our study suggests that tNGS can be a cost-effective tool for DR-TB diagnosis, particularly in settings where comprehensive drug susceptibility testing (DST) is not routinely performed. The findings underscore the importance of considering existing DST practices and healthcare infrastructure before implementing tNGS, especially in countries with varying levels of resource availability and TB burden. Future research should focus on further refining cost-effectiveness models and assessing the long-term impacts of integrating tNGS into TB control programs.

## Introduction

Drug-Resistant Tuberculosis (DR-TB) has become growing public health threat, with incidence increasing and only one third of people with multi-drug resistance (MDR)/Rifampicin resistant (RR) -TB diagnosed and enrolled in treatment annually.^1^ DR-TB is more difficult to diagnose as it requires bacteriological confirmation with drug susceptibility testing (DST). As novel agents and treatment regimens are introduced, there is also an emerging concern about resistance to two newer DR-TB drugs i.e. bedaquiline (BDQ) and linezolid (LZD).^2^^,^^3^

To end the global DR-TB epidemic, universal DST which includes new and repurposed TB drugs should be accessible. While WHO-recommended rapid molecular diagnostics (mWRDs) can detect resistance to rifampicin, follow-up testing by low complexity automated nucleic acid amplification tests (LCaNAATs), Line Probe Assays (LPA) or culture is typically required for detection of additional drug-resistance.^4^ Also, none of the mWRDs can detect resistance to new and repurposed drugs like bedaquiline, linezolid, delamanid and pretomanid.^5^ Culture-based methods are current standard of care for DST but this requires Biosafety Level 3 (BS3) laboratories; specialized reagents, cold-chain requirements; and may take 6–8 weeks for results, leading to delays and negative impacts on patient outcomes.^6^ Phenotypic DST (pDST) is also not widely available for newer drugs.^2^ An alternative DST method includes LPAs but this assay performs poorly on smear-negative and scanty samples.^3^^,^^4^^,^^7^ LPAs are also quite laboratory and staff intensive; requiring complex laboratory infrastructure and expensive equipment that is normally only available in reference laboratories.^8^ The LCaNAATs although simpler and faster than LPAs, these have not reached wide scale adoption and are currently recommended for a limited number of drugs including fluoroquinolones and isoniazid but none of the new drugs.

One promising DST option is targeted next-generation sequencing (tNGS), which amplifies selected genes with next-generation sequencing technology to detect resistance to many drugs with a single test.^9^ As tNGS can interrogate entire genes to identify specific mutations associated with resistance, it may provide improved accuracy compared with existing mWRDs.^10^ The other advantages of tNGS include improved speed, and comprehensive coverage of many more potential mutations.^11^ tNGS is valuable tool for the surveillance of DR-TB which allows the prediction of resistance to several anti-TB drugs including new and repurposed DR-TB drugs.^4^^,^^12^

Despite numerous advantages of tNGS, particularly in low and middle-income counties (LMICs) with a high burden of DR-TB, implementation of tNGS requires investment. TB programs globally continue to face inadequate funding, constraining available resources.^13^ Hence, it's crucial to ensure that decision making is informed through critical economic evidence concerning tNGS technologies. This study aimed to generate economic evidence to aid the World Health Organization (WHO) guideline development group meeting (GDG) on formulating guidance on the use of tNGS for the detection resistance to anti-TB drugs. The primary aim of this study was to assess the potential cost-effectiveness of introducing the tNGS for the detection of DR-TB in South Africa, Georgia, and India.

This study assessed the cost-effectiveness of tNGS in three different contexts. First, we evaluated the cost-effectiveness of tNGS as a test for DST among persons with RR TB compared to phenotypic DST. Second, we analyzed the cost-effectiveness of using tNGS as a test for DST among persons with RR TB, comparing it to in-country DST practices: Xpert MTB/XDR and pDST in South Africa and Georgia, and LPA and pDST in India. Finally, we assessed the cost-effectiveness of tNGS as an initial test for TB drug resistance in patients with bacteriologically confirmed TB, compared to the use of mWRDs followed by pDST in Georgia.

## Methods

### Study design

We developed stochastic decision analysis model to assess the cost-effectiveness of introducing tNGS for the diagnosis of DR-TB for all three objectives. This study was done from healthcare system perspective and accounted for healthcare system costs to diagnose and treat TB. The primary outcome of this study was incremental cost-effectiveness ratio (ICER) calculated as the incremental cost in USD per disability-adjusted life years (DALYs) averted. The ICER was computed by comparing the difference in costs and DALYs averted between the tNGS strategy and the comparator.

### Model structure, intervention, and comparator

#### Model structure

**Fig. 1:**
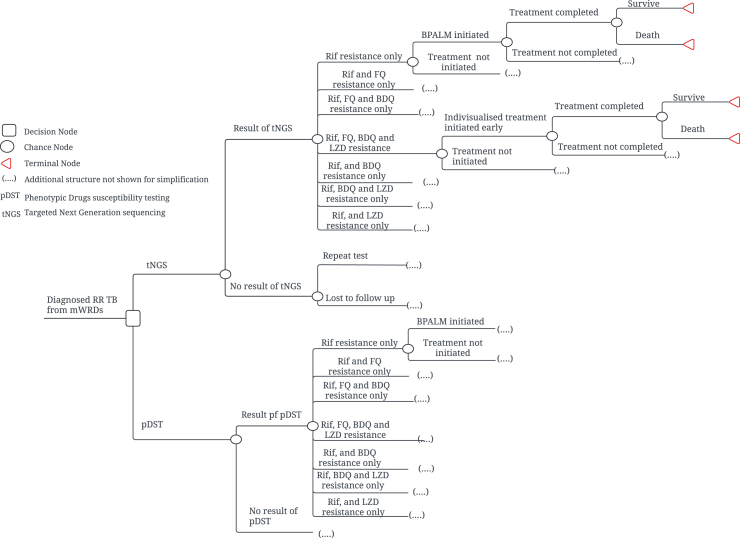
**Simplified model decision structure. Two strategies were compared: DST with tNGS versus pDST as a test for DST among person with RR.** Schematically these strategies are separated by a square representing a decision node. The circles represent chance nodes where individuals may experience one of several possible events shown on subsequent lines. The probabilities of developing each event are listed in Table 1. Dotted lines represent model structure omitted for simplicity. In all cases, this omitted structure parallels that shown. The diamond symbol represents terminal node. RR, Rifampicin resistance; tNGS, Targeted next-generation sequencing; pDST, Phenotypic Drugs susceptibility testing; Rif, Rifampicin; FQ, Fluroquinolone; BDQ, Bedaquiline; LZD, Linezolid; Rx., treatment; TB, Tuberculosis.

The decision analysis model employed a decision tree framework to simulate the diagnostic and treatment pathways for individuals with DR-TB. Each decision tree captured the sequence of testing, treatment decisions, and health outcomes, including true positive and false negative diagnostic results, treatment initiation based on DST outcomes, and final health outcomes such as survival and death. In our study, we used three different models to assess the cost effectiveness of tNGS. The first two models tracked individuals with RR when tNGS was used as test for DST among person with RR. First, model compared tNGS with universal DST (Fig. 1) and second model compared tNGS with in-country DST practice (Supplementary Figures S1 and S2). In both models, the probability of receiving test results depend on the indeterminant or contamination rate specific to tNGS and pDST, respectively. Within the model, DST was done only for group A DR-TB drugs (fluoroquinolone, bedaquiline and linezolid).^14^ We treated those individuals with rifampicin resistance as MDR TB and did not specifically consider DST for INH. The prevalence rate of fluoroquinolone (FQ) resistance, bedaquiline (BDQ) resistance and linezolid (LZD) were specific to each country.^15^, ^16^, ^17^, ^18^, ^19^ In the model we made an assumption that there was no resistance to other drugs outside Group A drugs used to treat DR TB largely due to limited or non-existent data regarding resistance to other DR-TB drugs. Once results were received, they either initiated or didn't initiate TB treatment ultimately resulting in either survival or death. Based on the DST results, treatment with Bedaquiline, Pretomanid, Linezolid and Moxifloxacin (BPALM), BPAL or an individualized regimen was initiated.^14^ Since there was no clinical or programmatic data on effectiveness of individualised regimen guided by pDST or tNGS, we assumed that once individuals were diagnosed to have BDQ and/or LZD resistance, in addition to RR, the individualized treatment would include a minimum of four effective drugs. In every model, loss to follow-up in both intervention and comparator is the same so as not to unduly favour tNGS. Model comparing tNGS with in-country DST in India, since BDQ DST was not routinely performed among RR-TB patients, we assigned higher mortality to BDQ-resistant individuals in the in-country DST arm, as they would be initiated on regimens including BDQ, resulting in fewer than four effective drugs.

The third model tracked hypothetical cohort of bacteriologically confirmed TB individuals in the high DRTB burden setting of Georgia. They were offered either tNGS or Xpert MTB/RIF followed by pDST as an initial DST (Supplementary Figure S3). Since tNGS can simultaneously detect resistance to several drugs with a single test, in the tNGS arm DST of Rif, INH, FQ, BDQ and LZD was done simultaneously. In the comparator arm, we assumed that only those with rif. resistance in mWRDs were further subjected to DST for FQ, BDQ and LZD through pDST. It was assumed that DST of INH among RIF sensitive individuals in the comparator arm was not done.

### Epidemiological, diagnostic and programmatic parameters

We gathered epidemiological parameters–prevalence data, diagnostic accuracy data and health outcome data–that were essential for the model from various sources. A detailed list of those sources is included in Table 1. The estimate and ranges for the prevalence of DR-TB were derived from the 2021 WHO DR-TB surveillance sheet, published DR survey report,^15^^,^^17^ published literature, and in consultation with WHO experts. Parameters on treatment initiation, completion and outcomes were taken from published literature for BPaLM, BPaL and individualized treatment, WHO guidelines and the 2022 global TB report.^14^^,^^19^^,^^32^^,^^33^ We used data from the systematic review on the diagnostic accuracy of tNGS done as a part of evidence generation for the WHO's GDG which covered the studies published until September of 2022 as well as the recently completed Foundation for Innovative New Diagnostics (FIND) multicenter clinical trial to assess the performance of culture-free, end-to-end tNGS solutions for diagnosis of DR-TB trial. The diagnostic accuracy of the comparator which included pDST, LPA and mWRDs was extracted from published literature. Utility data were sourced from the global burden of disease study.^46^

**Table 1:** Key model parameter estimate, ranges for sensitivity analysis and data sources.

Description	South Africa	India	Georgia	References
	Estimate	Estimate	Estimate	
**Prevalence data**				
Prevalence of FQ resistance among RR	0.13 (0.05–0.21)	0.3 (0.22–0.38)	0.29 (0.25–0.33)	15, 16, 17, 18
Prevalence of BDQ resistance among RR and FQR	0.17 (0.07–0.27)	0.15 (0.07–0.23)	0.15 (0.07–0.23)	^15^^,^^18^^,^^20^^,^^21^
Prevalence of BDQ resistance among RR and FQs	0.05 (0.02–0.08)	0.035 (0.01–0.06)	0.035 (0.01–0.06)	^20^^,^^22^, ^23^, ^24^, ^25^, ^26^, ^27^
Prevalence of LZD resistance among RR, FQR and BDQR	0.065 (0.03–0.10)	0.065 (0.03–0.1)	0.175 (0.1–0.25)	^15^^,^^18^
Prevalence of LZD resistance among RR, FQR and BDQs	0.026 (0.01–0.042)	0.035 (0.01–0.06)	0.04 (0.01–0.07)	^15^^,^^18^^,^^28^^,^^29^
Prevalence of LZD resistance among RR, FQs and BDQR	0.065 (0.03–0.10)	0.065 (0.03–0.1)	0.175 (0.1–0.25)	^27^^,^ Assumption
Prevalence of LZD resistance among RR, FQs and BDQs	0.026 (0.01–0.042)	0.0275 (0.01–0.045)	0.04 (0.01–0.07)	^21^^,^^30^^,^^31^
**Treatment outcome**^a^				
Probability of treatment initiation	0.9 (0.72–1)			^19^
Probability of completion of BPaLM/BPaL	0.9 (0.83–0.95)			^14^^,^^32^^,^^33^
Probability of Individualized treatment completion	0.73 (0.657–0.766)			^14^
Probability of death with BPaLM regimen	0.03 (0–0.6)			^14^^,^^32^^,^^33^
Probability of death with BPaL regimen	0.03 (0–0.6)			^14^^,^^33^^,^^34^
Probability of death with Individualized RX	0.125 (0.06–0.25)			^34^^,^^35^
Probability of death no treatment of DR TB (18 months)	1 (0.648–1)			^34^^,^ Assumption
Probability of death with wrong/incomplete treatment of DR TB (Annual)	0.486 (0.38–0.58)			^36^^,^^37^
Percentage of contamination and failure of growth in control tube pDST	0.13 (0.10–0.15)			Country data
Percentage of Indeterminate in tNGS	0.1 (0.08–0.13)			tNGS Diagnostic accuracy SR
Percentage of repeat test among contaminated samples in pDST and tNGS	0.9 (0.72–1)			Country data
**Cost data**				
Cost of BPaLM treatment	$3730 ($2984–$4476)	$953 ($762–$1143)	$3505 ($2804–$4206)	^38^
Cost of BPaL treatment	$3544 ($2835–$4252)	$918 ($734–$1101)	$3421 ($2736–$4105)	
Cost of Individualized treatment	$4787 ($3829–$5744)	$1036 ($829–$1243)	$4463 ($3570–$5535)	
Unit test cost tNGS	$197 ($134–$257)	$159 ($121–$175)	$166 ($120–$198)	tNGS CEA SR, FIND, Manufacturer
Unit test cost pDST	$95 ($68–$120)	$74 ($56–$92)	$83 ($57–$108)	39, 40, 41, 42
Unit test cost LPA	–	$103 ($60–$115)	–	
Unit test cost Xpert XDR	$39 (37–41)	–	$39 (37–41)	^43^^;^ Assumption
Unit test cost Xpert MTB/Rif	$29 (27–31)	$29 (27–31)	$29 (27–31)	^44^^,^^45^

Diagnostic accuracy data^a^	tNGS	pDST	LPA	
Rif				tNGS Diagnostics accuracy SR^51^, ^52^, ^53^, ^54^, ^55^
Sensitivity	0.96 (0.91–1)	0.982 (0.93–1)		
Specificity	0.94 (0.86–1)	0.81 (0.69–0.93)		
Inh				
Sensitivity	0.96 (0.93–0.99)	0.99 (0.94–1)	0.89 (0.89–0.92)	
Specificity	0.97 (0.95–0.99)	0.98 (0.95–0.99)	0.98 (0.97–0.99)	
FQ				
Sensitivity	0.97 (0.94–1)	0.99 (0.76–1)	0.93 (0.9–0.95)	
Specificity	0.95 (0.91–0.99)	1 (0.98–1)	0.99 (0.98–1)	
BDQ				
Sensitivity	0.68 (0.43–0.93)	1 (0.98–1)		
Specificity	0.97 (0.94–1)	1 (0.99–1)		
LZD				
Sensitivity	0.69 (0.39–0.99)	1 (0.51–1)		
Specificity	1	1 (0.96–1)		
**Utility data**^a^				
DALYs death	1			^46^^,^^56^
DALYs DR-TB individuals on treatment	0.33			
DALYs with no treatment/LTFU	0.66			

a Same for all 3 countries.

### Cost parameters

We only included costs incurred by the health care system (Table 1). Per unit test cost of tNGS were derived from a systematic review on the cost-effectiveness of tNGS and empirical costing done in consultation with manufacturers and FIND in preparation for the GDG meeting. The costs of the different treatment regimens for DRTB were extracted from a recently published cost-effectiveness study done by Sweeney et al. on short oral treatment regimens in the countries selected of this study.^38^ We assumed that the cost of individualized treatment will be same regardless of DST result. Per unit test costs of other diagnostics were taken from published literature and the Value TB studies which provide comprehensive cost information for various tuberculosis interventions.^47^ All costs were converted to 2021 using inflation rates in the respective local currency and then converted to 2021 USD using US bureau of labour statistics.

### Incremental cost-effectiveness

This study measured outcomes in terms of total costs and DALYs, with the primary economic measure being incremental cost per DALY averted. When tNGS was used as a test for DST among person with RR, we analyzed the incremental cost per DALY averted of introducing tNGS, compared to universal pDST and in-country DST practice. When tNGS was used as an initial test for TB drugs resistance, we analyzed the incremental cost per DALYs averted of introducing tNGS compared to Xpert MTB/RIF followed by pDST. A willingness to pay (WTP) threshold of 3 times the countries’ gross domestic product (GDP) per capita was employed as per World Bank report of 2021.^48^ This WTP threshold of 3 times GDP per capita was based on WHO-CHOICE^49^ recommendation and considering the significant health and economic benefits of effective DR-TB management.

### Statistics

Uncertainty around included parameters and the impact of this uncertainty on model results were explicitly examined through probabilistic sensitivity analyses (PSA). The primary outcome of ICER were obtained from a Monte Carlo simulation with 10,000 replications with 95% uncertainty ranges reported as the 2.5th and 97.5th percentiles of corresponding distributions.

### Sensitivity and scenario analysis

One-way sensitivity analysis was conducted to understand the potential impact of key model inputs on the ICER. We evaluated each individual parameter value independently. Parameters that showed greater influence, such as per unit test cost, contamination rate, repeat test probability (considered a proxy for loss to follow-up (LTFU)) were presented in tornado diagrams. Two-way sensitivity analysis was carried out on unit costs of tNGS and LTFU and presented graphically.

To evaluate the impact of various scenarios on cost-effectiveness, additional scenario analyses were performed. One scenario assessed the cost-effectiveness of tNGS compared to phenotypic DST, where individualized treatment in pDST arm lacked four effective drugs due to its limitation to perform DST of certain other drugs used to treat DR TB, leading to higher mortality. Other scenario analyses focused on cost-effectiveness of using tNGS for DST among persons with RR TB compared to in-country DST practice. We explored scenario that accounted for the benefit of using tNGS for multiple disease. A volume-based analysis accounting for the benefit of patient volume was also explored in the scenario analysis. We also did a scenario analysis with reduced tNGS test kit costs, assuming a 50% reduction in test kit price. A scenario with increased tNGS costs due to 20% fewer samples was also included. We conducted a scenario analysis where there was no LTFU in tNGS compared to 10% LFTU in pDST to give an advantage to tNGS for its faster turnaround time. We also conducted scenario analysis with increased BDQ resistant at 30%.

### Ethics

Ethical approval was obtained from the University of Ottawa Research Ethics Boards (H-07-22-8325–ANN1-8325). This analysis did not involve human subjects.

### Role of funding source

The funder itself had no role in the design, conduct, analysis or in the decision to submit for publication.

## Results

### Cost-effectiveness of tNGS versus universal pDST for DST among persons with RR

The cost-effectiveness results for using tNGS as a test for DST after detection of RR, replacing pDST without considering differences in time to results and potential impact on loss to follow-up is shown in Table 2. In this hypothetical context, the pDST approach resulted in fewer DALYs compared with tNGS and has lower costs across all three assessed countries. Under these conditions, pDST is considered dominant or preferred over tNGS.

**Table 2:** CEA result of using tNGS for DST.

Country	Comparator	DALYs	Cost	ICER ($ per DALY averted, 95% uncertainty ranges)
**Cost-effectiveness result when tNGS was used as a test for DST among persons with RR compared to universal pDST**				
South Africa	pDST	0.504	$3289	Ref.
	tNGS	0.508	$3404	Dominated by pDST (Dominated–$303,635)
Georgia	pDST	0.503	$3085	Ref.
	tNGS	0.508	$3183	Dominated by pDST (Dominated–$171,666)
India	pDST	0.503	$878	Ref.
	tNGS	0.508	$970	Dominated by pDST (Dominated–$162,897)
**Cost-effectiveness result when tNGS was used as a test for DST among persons with RR compared to in-country DST practice**				
South Africa	XpertXDR + pDST	0.519	$3223	Ref.
	tNGS	0.509	$3401	$15,619 (Cost saving-$114,782)
Georgia	XpertXDR + pDST	0.516	$3040	Ref.
	tNGS	0.508	$3183	$18,375 (Cost saving–$158,972)
India	LPA and pDST	0.5073	$981	Ref.
	tNGS	0.5072	$971	Dominates LPA and pDST (Cost saving–$60,083)
**Cost-effectiveness result when tNGS was used as an initial test for TB drug resistance in patients with bacteriologically confirmed TB**				
Georgia	Xpert MTB/Rif + pDST	0.51	$1180	Ref.
	tNGS	0.488	$1377	$9261 ($5258–$32,040)

DALY, Disability Adjusted Life Year; ICER, Incremental Cost-Effectiveness Ratio; tNGS, Targeted Next-Generation Sequencing; pDST, Phenotypic Drugs Susceptibility Test.

### Cost-effectiveness of tNGS versus in-country DST practice for DST among persons with RR

**Fig. 2:**
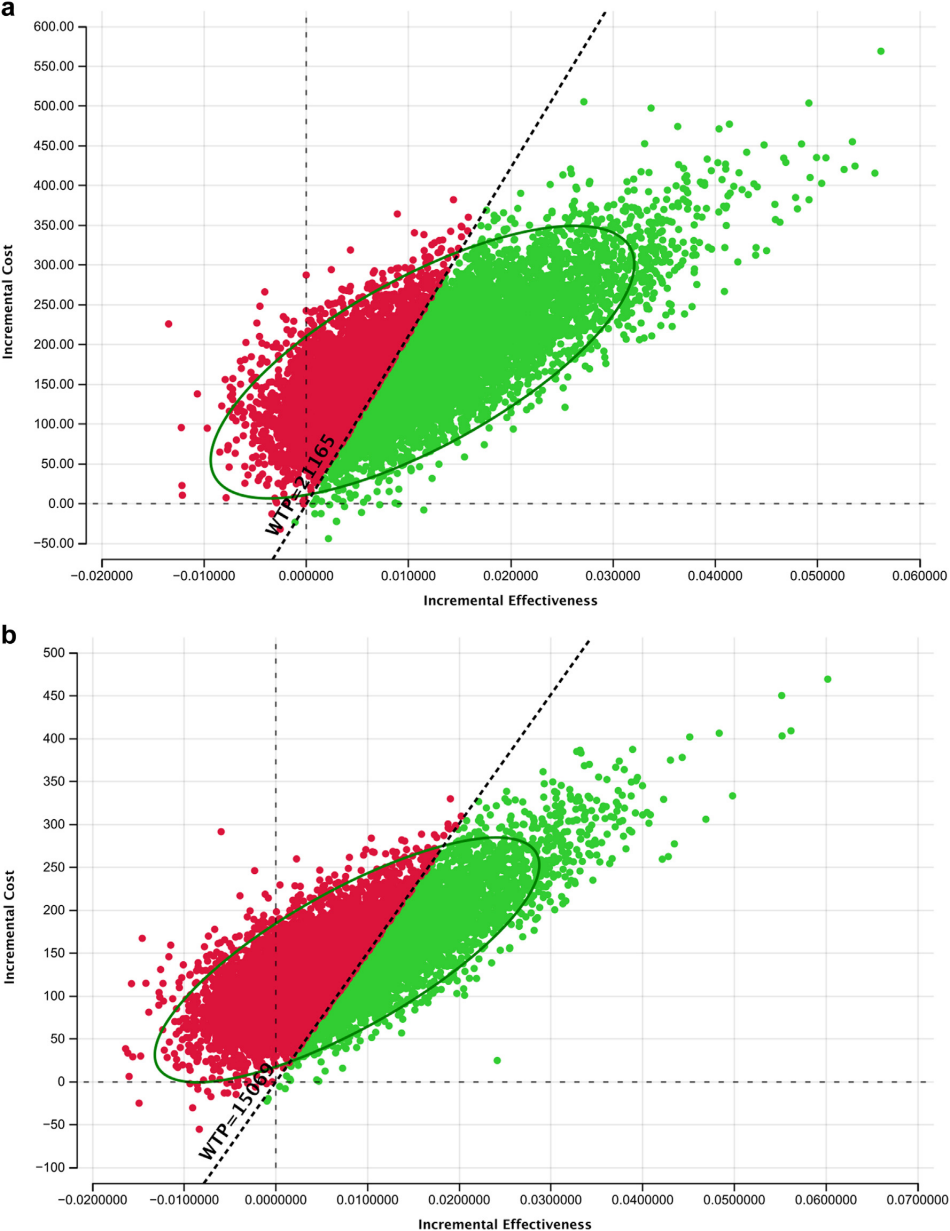

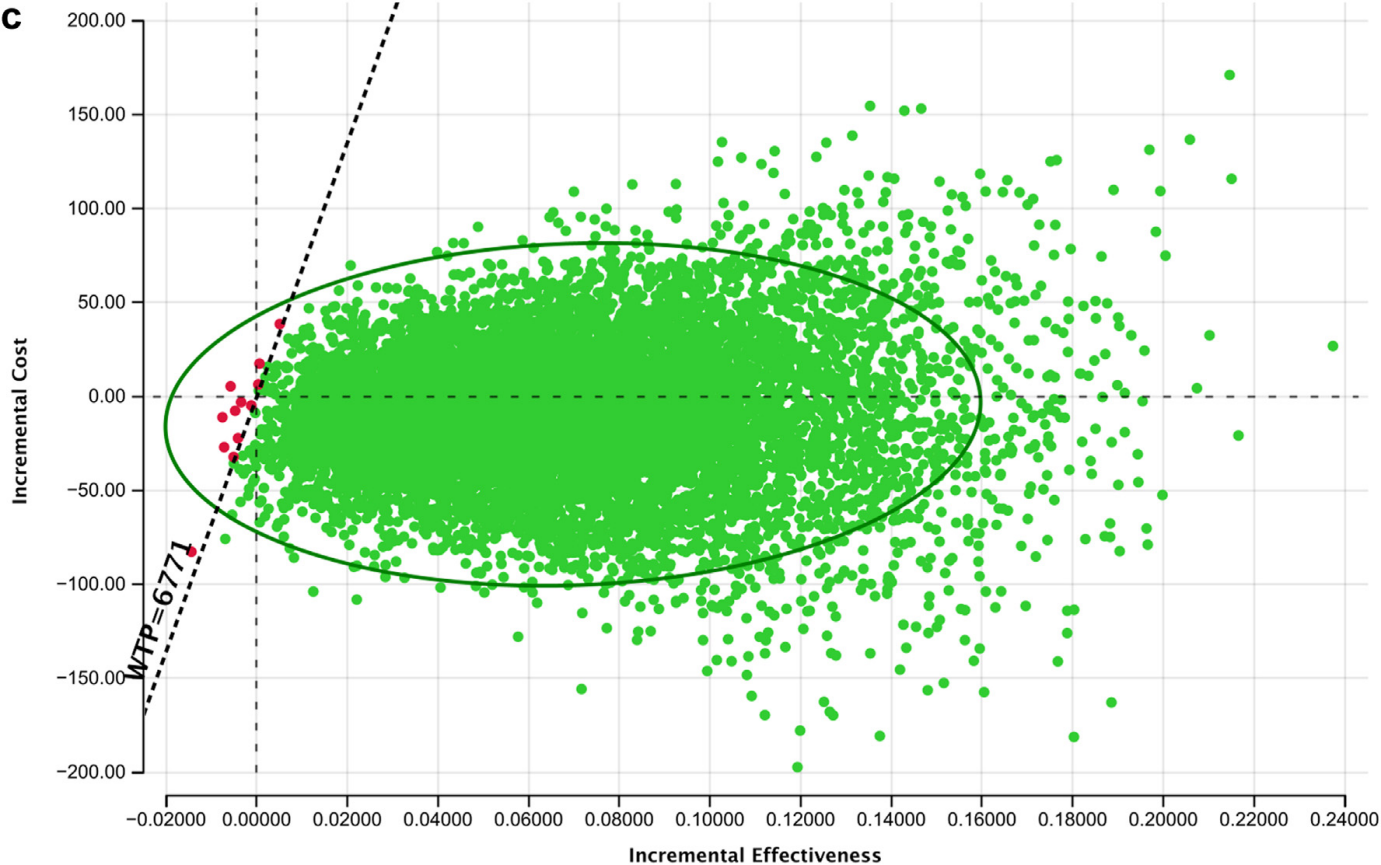
**a. Incremental cost-effectiveness scatterplot comparing tNGS versus South Africa's in-country DST as a test for DST among person with RR.** Each dot represents the result of iteration out of 10,000 Mont Carlo simulation. The dashed diagonal shows the willingness to pay threshold of $21,165 per DALY averted. The green DOTs that are below WTP threshold represent cost-effective simulations. **b: Incremental cost-effectiveness scatterplot comparing tNGS versus Georgia's in-country DST as a test for DST among person with RR.** Each dot represents the result of iteration out of 10,000 Mont Carlo simulation. The dashed diagonal shows the willingness to pay threshold of $15,069 per DALY averted. The green DOTs that are below WTP threshold represent cost-effective simulations. **c: Incremental cost-effectiveness scatterplot comparing tNGS versus India's in-country DST as a test for DST among person with RR.** Each dot represents the result of iteration out of 10,000 Mont Carlo simulation. The dashed diagonal shows the willingness to pay threshold of $6771 per DALY averted. The green DOTs that are below WTP threshold represent cost-effective simulations.

The incremental cost per DALYs averted of using tNGS as a test for DST after detection of RR, replacing in-country DST practice for South Africa and Georgia was $15,619 (95% UR: cost saving–$114,782) and $18,375 (95% UR: Cost saving–$158,972) respectively (Table 2). Using a WTP threshold at three times the country's GDP per capita, tNGS was cost effective in South Africa and not in Georgia. In India, tNGS dominated pDST leading to cost-savings and health gains compared to India's existing standard of care relying on pDST and LPAs. For South Africa and India, almost 60% of model iterations fell below the respective WTP thresholds whereas for Georgia only 40% of iterations fall below the WTP threshold (Fig. 2a–c).

### Cost-effectiveness of tNGS as an initial test for TB drug resistance

**Fig. 3:**
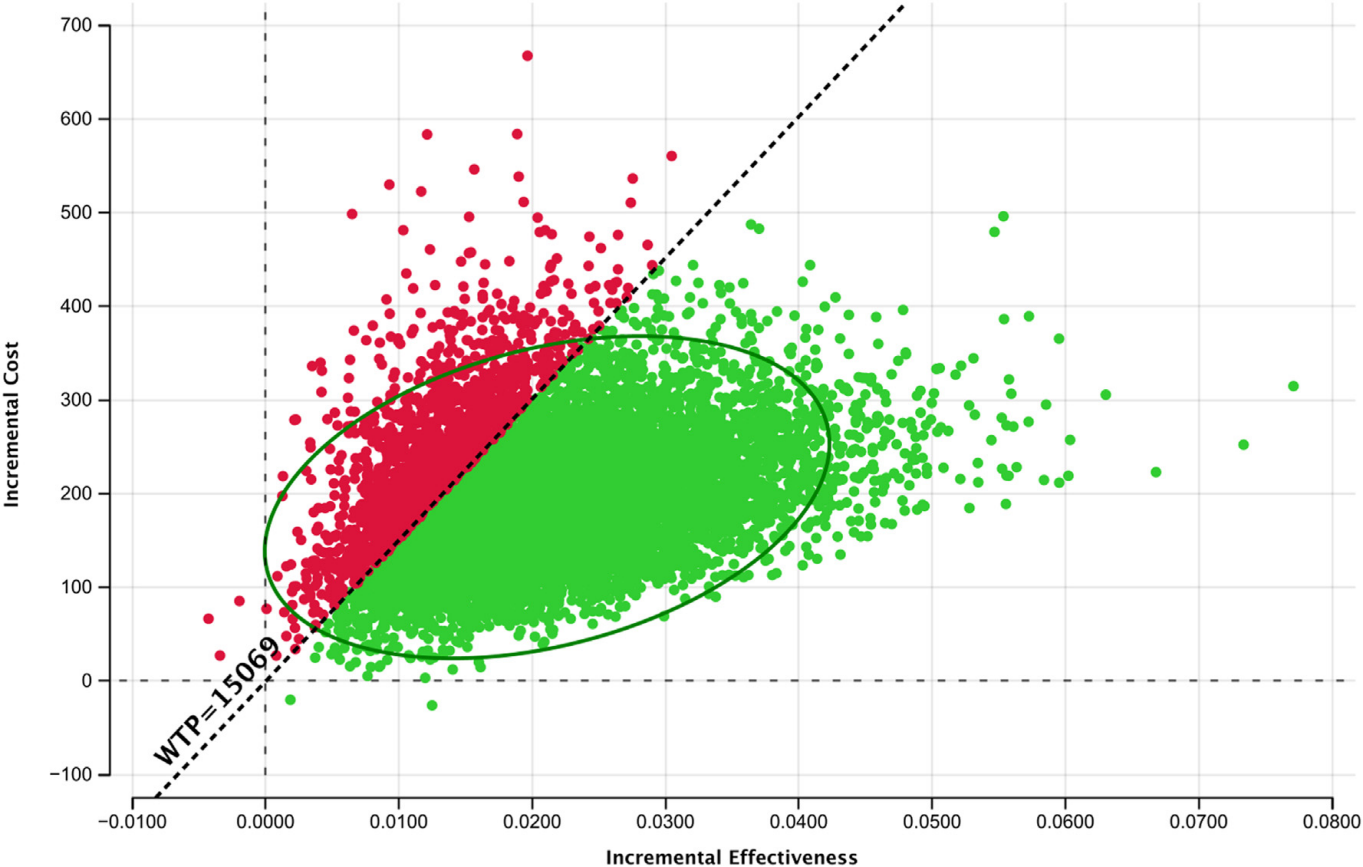
**Incremental cost-effectiveness scatterplot comparing tNGS as an initial test for TB drug resistance in patients with bacteriologically confirmed TB compared to use of mWRDs followed by pDST.** Each dot represents the result of iteration out of 10,000 Mont Carlo simulations. The dashed diagonal shows the willingness to pay threshold of $15,069 per DALY averted. The green DOTs that are below WTP threshold represent cost-effective simulations.

When tNGS was used as an initial test for TB drug resistance in the high DR TB setting of Georgia (Table 2) it resulted in improved health gains leading to an ICER of $9261 per DALY averted (95% UR: $5258–$32,040). At WTP threshold of 3 times the country's GDP using tNGS is cost-effective in high burden DR-TB setting of Georgia, with almost 70% of the iterations below the respective WTP threshold (Fig. 3).

### Sensitivity analysis

In univariable sensitivity analyses key epidemiological and cost parameters were varied across expected ranges to understand the influence of each parameter on model results. All variables having more than 10% change in model results were presented as a tornado diagram.

When tNGS was used as a test for DST among person with RR replacing pDST the most influencing parameter in the model for India and Georgia were the rate of probability of repeat testing among pDST and tNGS and per unit cost of tNGS. Conversely, for South Africa unit cost of tNGS and pDST exerted a greater effect on model outcome (Supplement Figure S4).

**Fig. 4:**
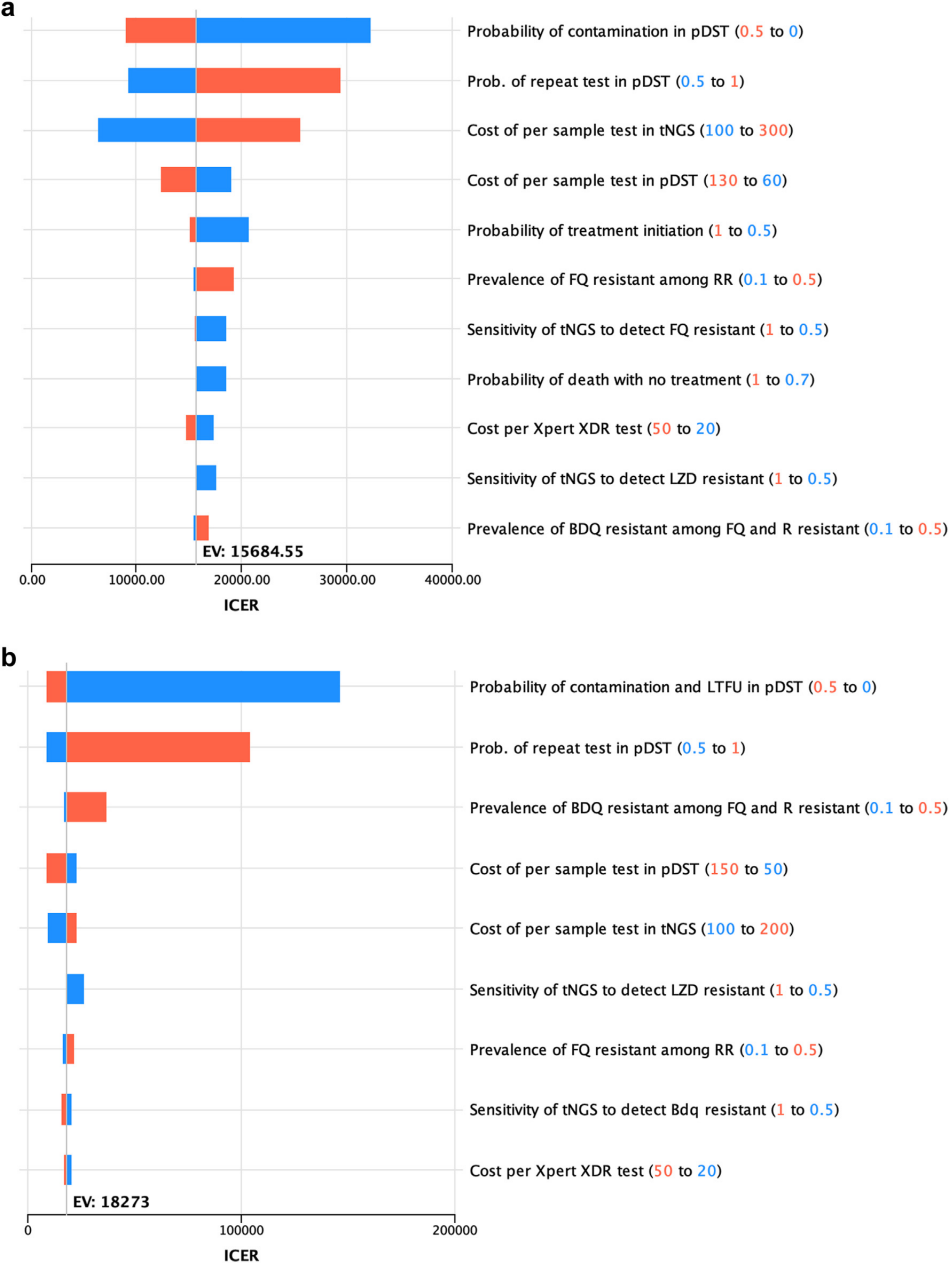

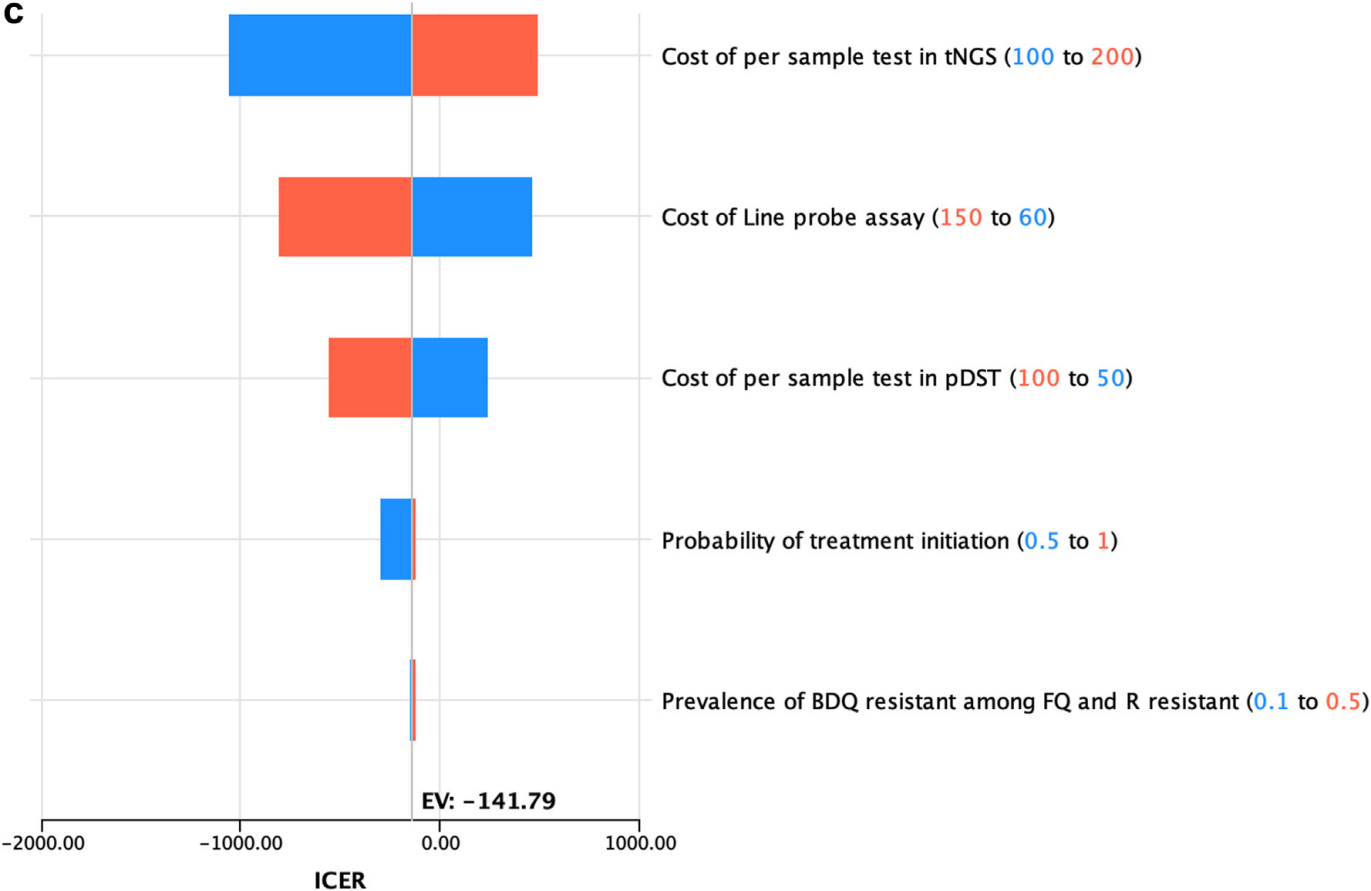
**a. One way sensitivity analyses tornado diagram on incremental cost-effectiveness ratio (ICER) of using tNGS as a replacement of South Africa's DST practice as a test for DST among person with RR.** In this diagram, the horizontal bars illustrate how the ICER varies as each parameter is adjusted individually within the specified range, while all other parameters are held constant. The length of each bar reflects the degree of influence that parameter has on the ICER, with longer bars indicating greater impact. The orange bars represent the ICER when the parameter is at its high value, and the blue bars represent the ICER when the parameter is at its low value. The parameters are listed in descending order of their impact on the ICER, from top to bottom. **b: One way sensitivity analyses tornado diagram on incremental-cost effectiveness ratio (ICER) of using tNGS as a replacement of Georgia's DST practice as a test for DST among person with RR.** In this diagram, the horizontal bars illustrate how the ICER varies as each parameter is adjusted individually within the specified range, while all other parameters are held constant. The length of each bar reflects the degree of influence that parameter has on the ICER, with longer bars indicating greater impact. The orange bars represent the ICER when the parameter is at its high value, and the blue bars represent the ICER when the parameter is at its low value. The parameters are listed in descending order of their impact on the ICER, from top to bottom. **c: One way sensitivity analyses tornado diagram on incremental cost-effectiveness ratio (ICER) of using tNGS as a replacement of India's DST practice as a test for DST among person with RR.** In this diagram, the horizontal bars illustrate how the ICER varies as each parameter is adjusted individually within the specified range, while all other parameters are held constant. The length of each bar reflects the degree of influence that parameter has on the ICER, with longer bars indicating greater impact. The orange bars represent the ICER when the parameter is at its high value, and the blue bars represent the ICER when the parameter is at its low value. The parameters are listed in descending order of their impact on the ICER, from top to bottom.

When tNGS was used as a test for DST among person with RR replacing in-country DST practice for South Africa and Georgia the rate of contamination of pDST, probability of repeat testing among pDST (proxy for LTFU) and per unit cost of tNGS exerted the greatest impact on ICER values (Fig. 4a–b). In India, the unit cost of tNGS, LPA and pDST were more influencing on model outcome (Fig. 4c). We have investigated these influencing parameters further in scenario analysis.

**Fig. 5:**
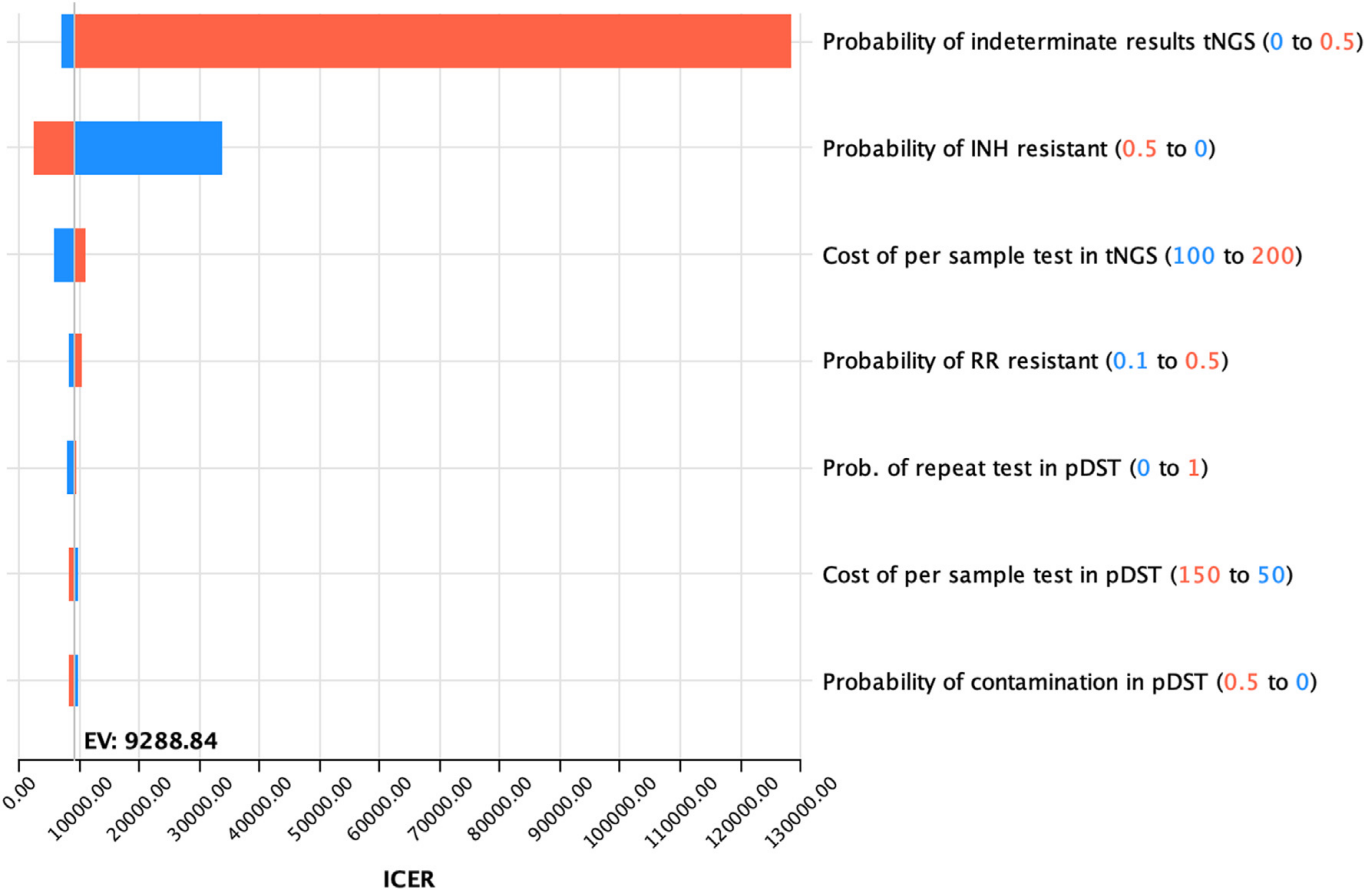
**One way sensitivity analyses tornado diagram on incremental cost-effectiveness ratio (ICER) of using tNGS as initial test for DR diagnosis.** In this diagram, the horizontal bars illustrate how the ICER varies as each parameter is adjusted individually within the specified range, while all other parameters are held constant. The length of each bar reflects the degree of influence that parameter has on the ICER, with longer bars indicating greater impact. The orange bars represent the ICER when the parameter is at its high value, and the blue bars represent the ICER when the parameter is at its low value. The parameters are listed in descending order of their impact on the ICER, from top to bottom.

When tNGS was used as the initial test for TB drugs resistance detection in the high DR TB burden setting of Georgia the rate of indeterminant result of tNGS, probability of INH resistance and per unit cost of tNGS had the largest influence on ICER values (Fig. 5). Cost effectiveness threshold based on prevalence of INH resistance and tNGS indeterminate rate was also done which is shown in supplementary article.

### Scenario analysis

Table 3 shows the result of scenario analyses done to evaluate cost effectiveness of using tNGS. When tNTS was compared to pDST, the scenario where patients in the pDST arm were initiated on the least effective individualized treatment, compared to tNGS, pDST was no longer dominant over tNGS. In the scenario where tNGS was employed for multiple diseases, cost-effectiveness improved across all three countries, with ICER per DALY averted of $12,530 (95% UR: cost saving-$86,202) and $15,808 (95% UR: cost saving- $137,387) in South Africa and Georgia, respectively. In India, utilizing tNGS for multiple diseases dominated the in-country DST practice. In scenario where there was 50% reduction in tNGS test kit cost resulted in tNGS to be cost-effective in all 3 countries. In South Africa and Georgia, the ICER per DALY averted was $12,757 (95% UR: cost saving–$79,122) and $14,077 (95% UR: cost saving–$112,771) whereas in India tNGS dominated the in-country DST practice. We also looked in the scenario where there is 20% fewer samples per run of tNGS due to low volume of samples received, it resulted in increased ICER per DALY averted and resulted in tNGS being only cost-effective in South Africa (ICER: $17,763/DALY averted, WTP: $21,165) but not in Georgia (ICER: $20,130/DALY averted, WTP: $15,069) and India (ICER: $7520/DALY averted, WTP: $6771). In scenario where there is no LTFU in tNGS due to early turnaround time resulted in tNGS to be cost-effective in South Africa (ICER: $13,004/DALY averted, WTP: $21,165) and Georgia (ICER: $13,640/DALY averted, WTP: $15,069) whereas in India tNGS dominated in-country DST practice. In scenario with high BDQ resistant the ICER per DALY averted was $16,144 and $22,889 for South Africa and Georgia respectively whereas in India tNGS dominated in-county DST practice. In Supplementary Table S1 we have additional scenario analysis result where the probability of death among untreated is 70%.

**Table 3:** Scenario analysis tNGS compared to pDST and in country DST practice among RR individuals.

Country	Comparator	DALYs	Cost	ICER ($ per DALY averted, 95% uncertainty ranges)
**Increased mortality among individual initiated in individualized treatment in pDST arm**				
South Africa	pDST	0.510	$3237	Ref.
	tNGS	0.508	$3404	$103,195 (Cost saving–$655,752)
Georgia	pDST	0.513	$3031	Ref.
	tNGS	0.508	$3183	$29,961 (Cost saving–$284,646)
India	pDST	0.511	$872	Ref.
	tNGS	0.508	$970	$23,065 (Cost saving–$241,534)
**Multiuse for multi-disease with reduced tNGS cost**^a^				
South Africa	XpertXDR + pDST	0.52	$3224	Ref.
	tNGS	0.509	$3367	$12,530 (Cost saving–$86,202)
Georgia	XpertXDR + pDST	0.516	$3038	Ref.
	tNGS	0.508	$3162	$15,808 (Cost saving–$137,387)
India	LPA and pDST	0.5076	$981	Ref.
	tNGS	0.5075	$961	Dominates LPA and pDST (Cost saving–$55,713)
**Reduced tNGS kit cost (50% price reduction)**^a^				
South Africa	XpertXDR + pDST	0.52	$3222	Ref.
	tNGS	0.508	$3368	$12,757 (Cost saving–$79,122)
Georgia	XpertXDR + pDST	0.516	$3043	Ref.
	tNGS	0.508	$3157	$14,077 (Cost saving–$112,771)
India	LPA and pDST	0.5074	$982	Ref.
	tNGS	0.5073	$939	Dominates (Cost saving–$22)
**20% fewer samples and increased unit test cost**^a^				
South Africa	XpertXDR + pDST	0.52	$3222	Ref.
	tNGS	0.508	$3421	$17,763 (Cost saving–$152,515)
Georgia	XpertXDR + pDST	0.516	$3040	Ref.
	tNGS	0.508	$3133	$20,130 (Cost saving–$185,104)
India	LPA and pDST	0.5075	$980	Ref.
	tNGS	0.5073	$981	$7520 (Cost saving–$70,187)
**Reduced LTFU in tNGS (0% versus 10%)**^a^				
South Africa	XpertXDR + pDST	0.52	$3223	Ref.
	tNGS	0.504	$3435	$13,004 ($7075–$44,119)
Georgia	XpertXDR + pDST	0.516	$3039	Ref.
	tNGS	0.503	$3212	$13,640 (Cost saving-$91,130)
India	LPA and pDST	0.507	$982	Ref.
	tNGS	0.502	$981	Dominates LPA and pDST (Cost saving–$48,801)
**80% sensitivity of tNGS to BDQ and LZD**^a^				
South Africa	XpertXDR + pDST	0.52	$3226	Ref.
	tNGS	0.508	$3406	$15,758 (Cost saving–$114,193)
Georgia	XpertXDR + pDST	0.516	$3040	Ref.
	tNGS	0.507	$3186	$17,124 (Cost saving–$176,728)
India	LPA and pDST	0.508	$981	Ref.
	tNGS	0.507	$971	Dominates LPA and pDST (Cost saving–$53,695)
**Increasing BDQ resistant at 30%**^a^				
South Africa	XpertXDR + pDST	0.52	$3234	Ref.
	tNGS	0.51	$3408	$16,144 (Cost saving–$128,456)
Georgia	XpertXDR + pDST	0.519	$3062	Ref.
	tNGS	0.513	$3199	$22,889 (Cost saving–$186,593)
India	LPA and pDST	0.584	$981	Ref.
	tNGS	0.513	$971	Dominates LPA and pDST (Cost saving–$911)

a These scenarios compared use of tNGS as test for DST among person with RR replacing in-country DST practice.

## Discussion

In this economic evaluation, we evaluated the cost-effectiveness of using tNGS for DST and detection of DR TB in the settings of South Africa, Georgia and India. When tNGS was compared with universal pDST, for DST among people with RR and assuming a similar probability of LTFU between tNGS and pDST, our findings indicated that tNGS was dominated by pDST. Therefore, in countries where pDST is well established without prohibitively high rates of LTFU, tNGS is less likely to be cost-effective. It should be noted that this is an idealistic comparator, as universal DST is not current practice is most countries particularly among low- and middle-income countries. Price of testing also influenced the overall cost-effectiveness and based on a 50% reduction which is expected upon market launch, tNGS would be cost-effective in all three settings.

When tNGS was compared as a replacement for current in-country DST practice, for DST among people with RR, tNGS was found to be cost-effective in South Africa. However, in Georgia, tNGS was not considered cost-effective according to WTP threshold of the country. In India, where the country is currently using LPA and pDST, tNGS dominated India's DST practice with lower costs and greater health gains. In India, BDQ pDST was conducted only among those who failed the BDQ regimen, and in South Africa, DST of BDQ and LZD is not being performed among FQ sensitive, though that is in the process of change. So, countries where universal DST of new and repurposed drugs is not being done routinely will benefit more from tNGS implementation. All three countries are currently using two DST methods after RIF. resistance. In South Africa and Georgia, they are using Xpert XDR followed by pDST and in India, they are using LPA and pDST. Therefore, countries with more complex and expensive existing DST algorithms (e.g. LPAs) are more likely to find tNGS cost-effective.

We found that tNGS was cost-effective as the initial test for DST after bacteriological confirmation of TB compared to mWRDs followed by pDST for those where RR is detected. Therefore, in countries with a high prevalence of DR TB and where DST of INH among those individuals with Rif. sensitive is not being done, implementing tNGS as the initial test for DST may be beneficial compared to mWRDs followed by pDST.

Different scenarios were looked at to assess the cost-effectiveness of using tNGS as a test for DST among person with RR replacing in-country DST practice. In the scenario where tNGS was compared to pDST, with pDST containing the least effective four drugs in the individualized treatment regimen due to its limitations in conducting DST for all other drugs, pDST was no longer dominant. This result shows that tNGS is more effective compared to pDST in scenarios where pDST cannot test for a wider range of drugs, leading to less effective treatment regimens and higher mortality. In the scenario where tNGS is used with a multi-disease testing approach, cost-effectiveness improved in all three countries suggesting that tNGS used for TB and other diseases like SARS-CoV2, HIV and cancers, may be beneficial and improve cost-effectiveness. When LTFU decreased with tNGS compared to the in-country DST practice in scenario analysis, cost-effectiveness improved. This suggests that introducing tNGS in countries experiencing high LTFU rates due to delayed DST results is more likely to be cost-effective. It implies placement at decentralized sites or centralized testing with highly efficient sample referral to achieve faster turnaround times. However, testing in either situation is likely to require batching need to achieve efficiency and decrease test cost but may increase the turnaround time and which might increase the chance of LTFU among DR-TB individuals. If batching is implemented only factoring in improvement to turnaround time, it will increase unit test cost and will also decrease likelihood of being cost-effective which was seen in the scenario with tNGS running with 20% fewer samples and was only cost-effective in South Africa since WTP threshold is higher in South Africa compared to Georgia and India.

To our knowledge, this is the first economic analysis assessing the cost-effectiveness of using tNGS for the detection of DR TB in LMICs. There are few limitations in the study underpinned by the lack of patient important outcomes using tNGS for DST. The first limitation is that the model did not account for disease transmission and the long-term impact of using tNGS in TB incidence, which might have underrepresented the cost-effectiveness of tNGS. When transmission-dynamic modelling was done using WGS DST by Mugwagwa et al. using WGS was initially associated with increase in annual cost but averting TB transmission and future TB cases subsequently resulted in cost savings and health benefits.^50^ In the model impact of faster turnaround time was incorporated into the model through its effect on LTFU rates, We acknowledge that this approach may not fully capture the potential benefits of tNGS in reducing morbidity, and this is a limitation of our current model. tNGS can simultaneously detect resistance to several drugs with a single test. Yet, in the model, we only included DST of INH and RIF among the first-line drugs. Not including PZA and EMB in this model might have underrepresented the potential cost-effectiveness of tNGS. The cost-effectiveness result of tNGS for DST after rapid molecular test for RIF only looked at DST of three group A drugs i.e. FQ, BDQ and LZD. Not including other drugs used in the treatment of DR-TB in this model with tNGS can provide results that might have also lessened the potential cost-effectiveness of tNGS. In the cost-effective result for tNGS as initial diagnostic, only those with resistance to RIF from mWRD were further subjected to pDST in the comparator arm. So, this result may not be generalized to the countries where DST to INH is being done among all bacteriologically confirmed pulmonary TB. This study used utility data from the Global Burden of Disease study, which does not focus specifically on DR-TB and may have underestimated its unique morbidity profile and health-related burden.

Implementing tNGS as an initial test for drug resistance detection in patients with bacteriologically confirmed TB may be cost-effective in a country with a high prevalence of DR TB such as Georgia, and where DST of INH and Group A second-line drugs is not being performed universally. If the country is considering implementing tNGS after rapid molecular testing for RIF resistance, then the cost-effectiveness of tNGS depends on the existing DST practice of the country. In countries where pDST is well established and provides universal DST of new and repurposed DR-TB drugs among individuals with RIF Resistance, implementing tNGS is less likely to be cost-effective. In contrast, in countries where universal DST of new and repurposed DR-TB drugs among RIF resistant individuals are not being done routinely, compared to their in-country DST practice implementing tNGS will be cost effective. In a scenario where tNGS can be used for multiple disease which will increase its utility and reduce cost implementing tNGS will be beneficial and cost-effective. Countries with high LTFU among RIF resistant individuals due to delayed follow-on DST results, the implementation of tNGS will more likely be cost-effective. In conclusion, implementing tNGS can be cost-effective depending on current DST practice and coverage. It is therefore important to consider the existing standard of care before implementation and scale-up of tNGS.

## Contributors

AZ and SS contributed to conceptualization of the project, data curation, modelling, formal analysis, and manuscript writing. In addition, AZ also provided supervision of overall project. CM and NI contributed to conceptualization of the project, and manuscript writing. AA contributed to data curation and analysis. The underlying data were verified by AZ and SKS. All authors read and approved the manuscript. AZ accepts full responsibility for the work and/or the conduct of the study, had access to the data, and decision to publish.

## Data sharing statement

The authors confirm that all parameters used in the cost-effectiveness analysis are available within the article and its Supplementary Materials. No new data were generated for this study.

## Declaration of interests

CM reports receiving funding from USAID to support the Global TB Programme staff at the WHO. All other authors declare no conflicts of interest.
