# Supplementary material for: Cost-effectiveness of targeted next-generation sequencing (tNGS) for detection of tuberculosis drug resistance in India, South Africa and Georgia: a modeling analysis
Source: eClinicalMedicine. 2024 Dec 24;79:103003. doi: 10.1016/j.eclinm.2024.103003 (PMC11732181; doi:10.1016/j.eclinm.2024.103003)
Supplement: Supplementary material [file mmc1.docx]

**Supplement**

**Supplement figures of additional model structure**


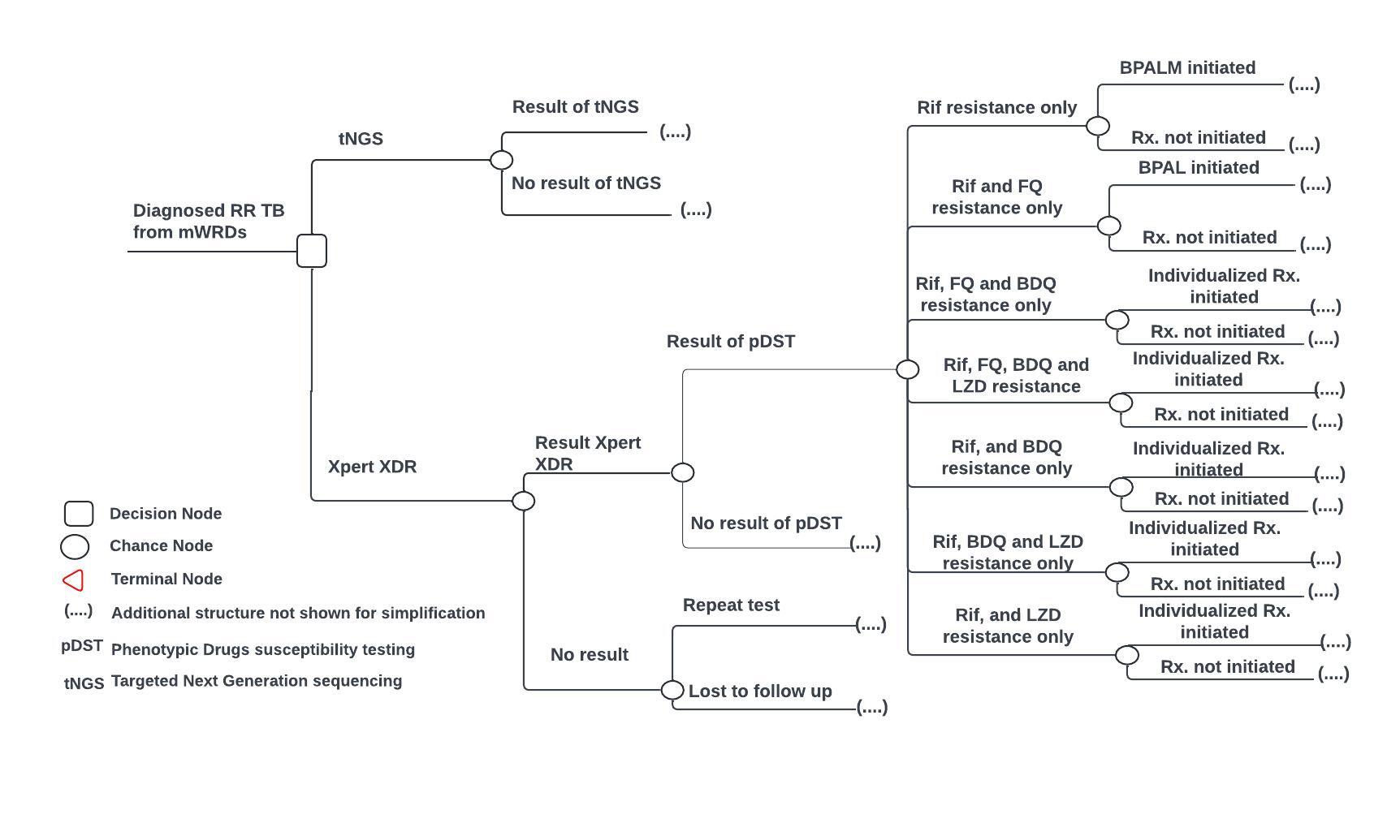


**Supplement Figure 1 :** Simplified model decision structure. Two strategies were compared: DST with tNGS vs South Africa and Georgia’s in country DST practice as a subsequent test for DST following detection of RR. Schematically these strategies are separated by a square representing a decision node. The circles represent chance nodes where individuals may experience one of several possible events shown on subsequent lines. The probabilities of developing each event are listed in Table 1. Dotted lines represent model structure omitted for simplicity. In all cases, this omitted structure parallels that shown. The diamond symbol represents terminal node. RR= Rifampicin resistance; tNGS=Targeted Next Generation sequencing; pDST= Phenotypic Drugs susceptibility testing; Rif=Rifampicin, FQ= Fluroquinolone; BDQ= Bedaquiline; LZD=Linezolid; Rx.=treatment, TB-Tuberculosis


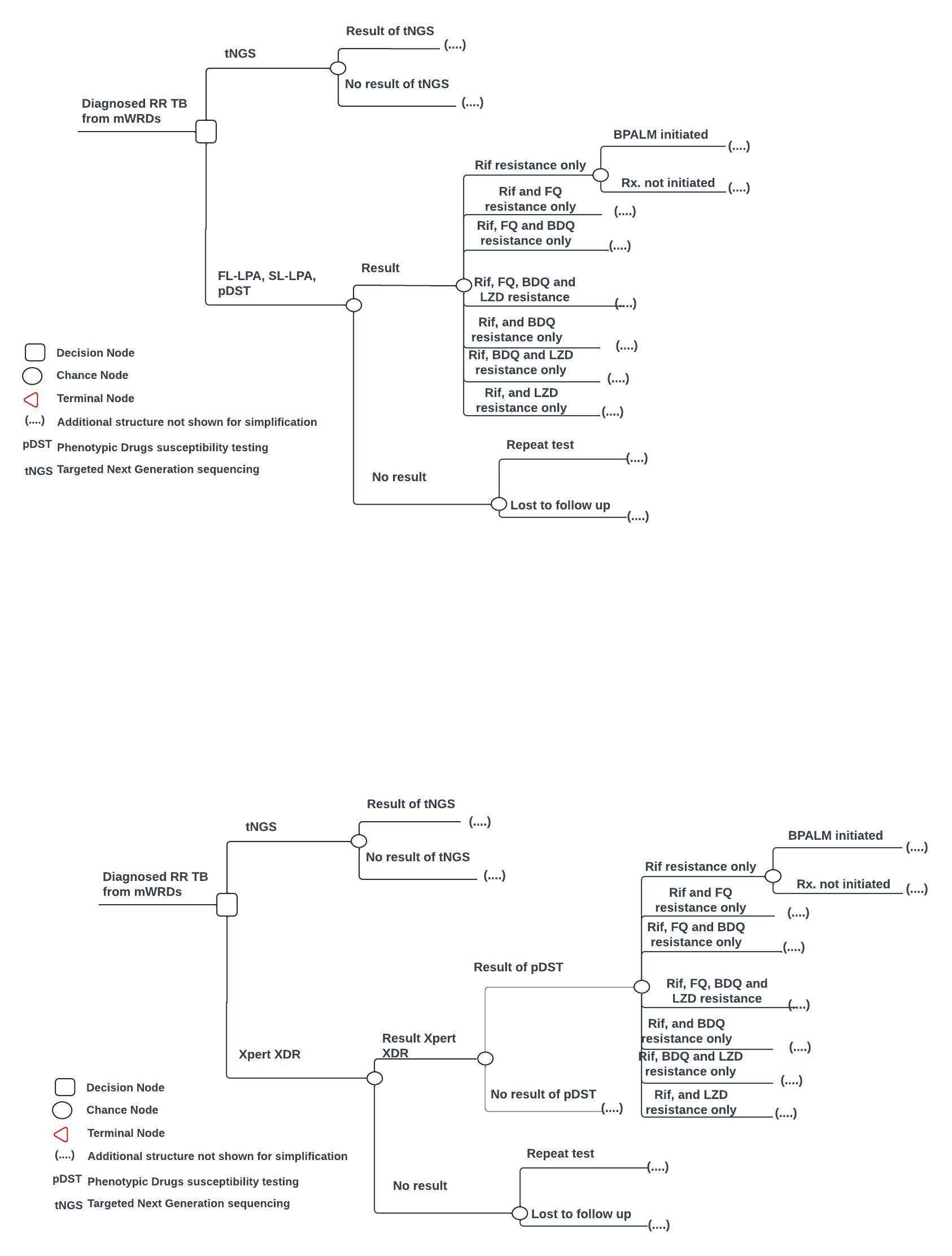


**Supplement Figure 2:** Simplified model decision structure*.* Two strategies were compared: DST with tNGS vs India’s in country DST practice as a subsequent test for DST following detection of RR. Schematically these strategies are separated by a square representing a decision node. The circles represent chance nodes where individuals may experience one of several possible events shown on subsequent lines. The probabilities of developing each event are listed in Table 1. Dotted lines represent model structure omitted for simplicity. In all cases, this omitted structure parallels that shown. The diamond symbol represents terminal node. RR= Rifampicin resistance; tNGS=Targeted Next Generation sequencing; pDST= Phenotypic Drugs susceptibility testing; Rif=Rifampicin, FQ= Fluroquinolone; BDQ= Bedaquiline; LZD=Linezolid; Rx.=treatment, TB-Tuberculosis ; LPA= Line Probe As


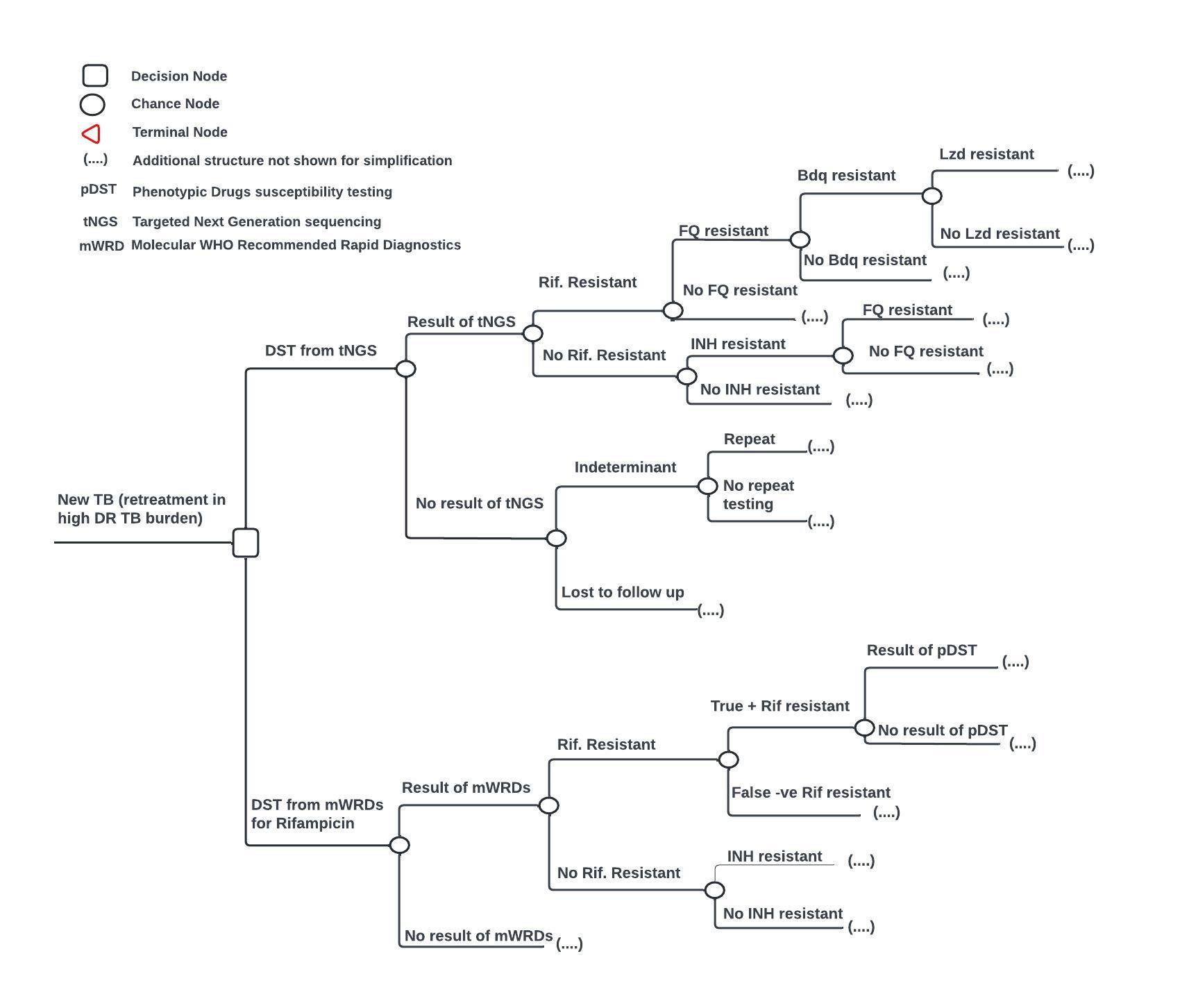


**Supplement Figure 3:** Simplified model decision structure*.* Two strategies were compared: DST with tNGS vs mWRDs followed by pDST among individuals with bac. confirmed TB. Schematically these strategies are separated by a square representing a decision node. The circles represent chance nodes where individuals may experience one of several possible events shown on subsequent lines. The probabilities of developing each event are listed in Table 1. Dotted lines represent model structure omitted for simplicity. In all cases, this omitted structure parallels that shown. The diamond symbol represents terminal node. RR= Rifampicin resistance; tNGS=Targeted Next Generation sequencing; pDST= Phenotypic Drugs susceptibility testing; Rif=Rifampicin, FQ= Fluroquinolone; BDQ= Bedaquiline; LZD=Linezolid; Rx. =treatment, TB-Tuberculosis; mWRD= Molecular WHO recommended Rapid Diagnostics

**Results supplement**

**Supplement figure 4: One way sensitivity of Objective 1**

**
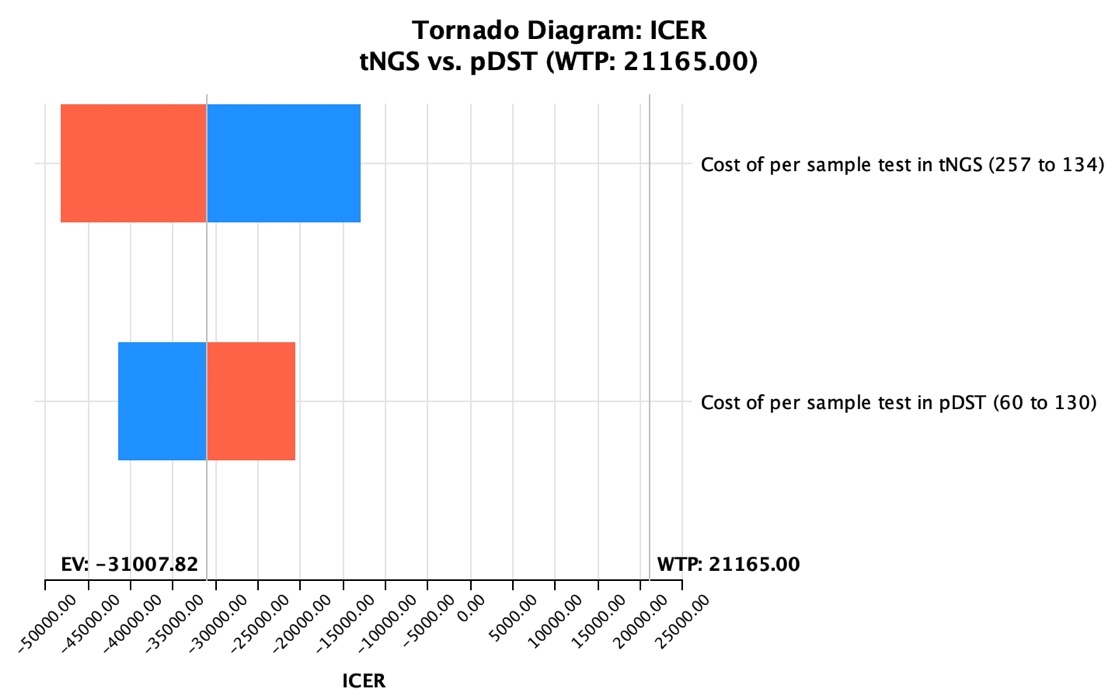
**

**Figure 4A:** One way sensitivity analyses tornado diagram on incremental cost effectiveness ratio (ICER) of using tNGS as a replacement of pDST in South Africa as a subsequent test for DST following detection of RR. In this diagram, the horizontal bars illustrate how the ICER varies as each parameter is adjusted individually within the specified range, while all other parameters are held constant. The length of each bar reflects the degree of influence that parameter has on the ICER, with longer bars indicating greater impact. The orange bars represent the ICER when the parameter is at its high value, and the blue bars represent the ICER when the parameter is at its low value. The parameters are listed in descending order of their impact on the ICER, from top to bottom.

**
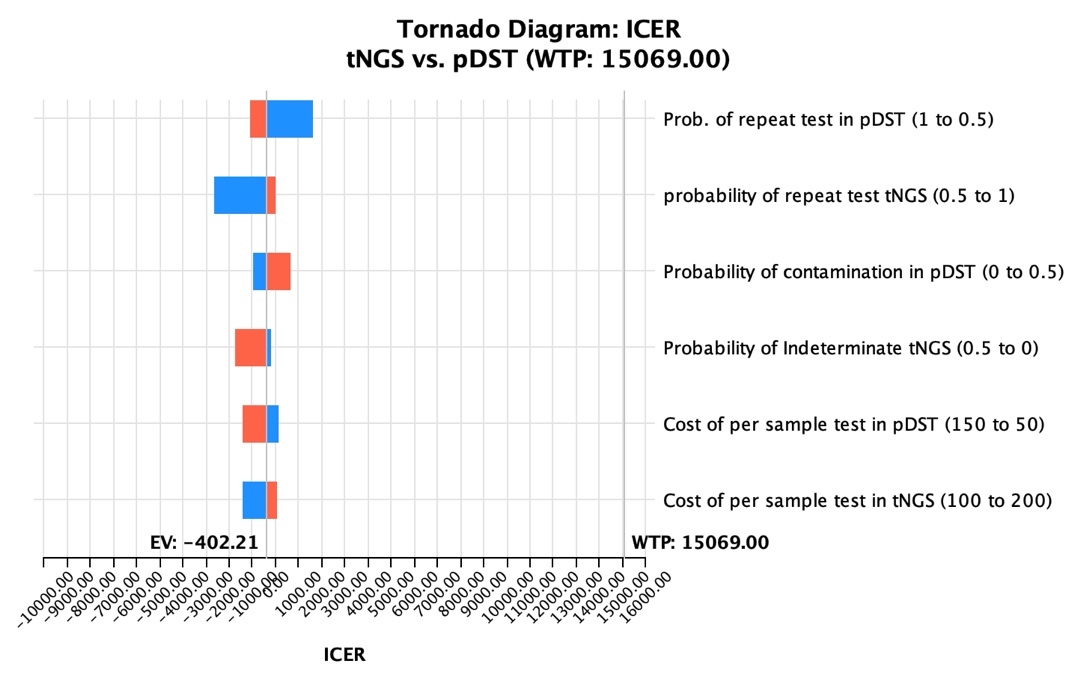
**

**Figure 4B:** One way sensitivity analyses tornado diagram on incremental cost effectiveness ratio (ICER) of using tNGS as a replacement of pDST in Georgia as a subsequent test for DST following detection of RR. In this diagram, the horizontal bars illustrate how the ICER varies as each parameter is adjusted individually within the specified range, while all other parameters are held constant. The length of each bar reflects the degree of influence that parameter has on the ICER, with longer bars indicating greater impact. The orange bars represent the ICER when the parameter is at its high value, and the blue bars represent the ICER when the parameter is at its low value. The parameters are listed in descending order of their impact on the ICER, from top to bottom.

**
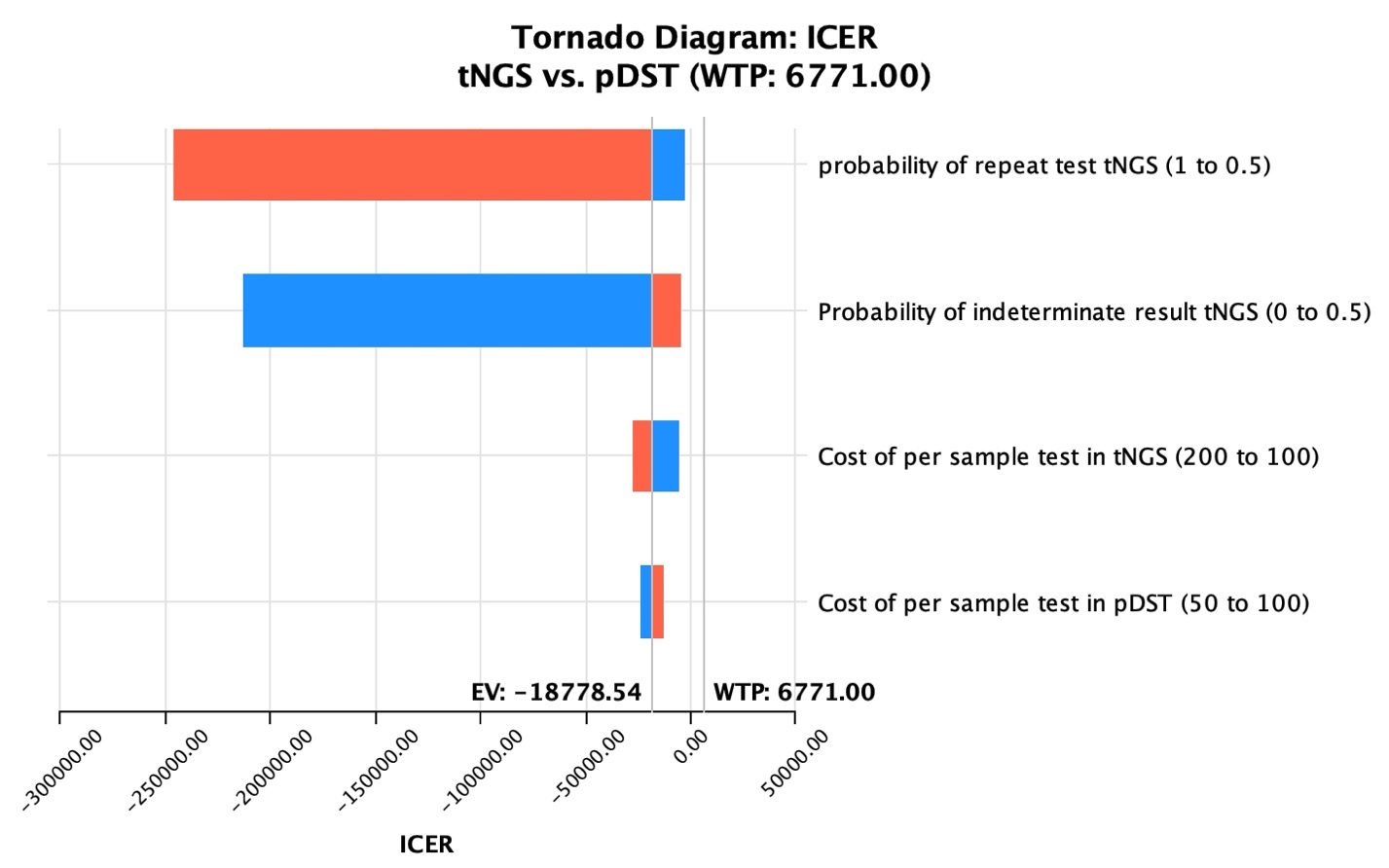
**

**Figure 4C:** One way sensitivity analyses tornado diagram on incremental cost effectiveness ratio (ICER) of using tNGS as a replacement of pDST in India as a subsequent test for DST following detection of RR. In this diagram, the horizontal bars illustrate how the ICER varies as each parameter is adjusted individually within the specified range, while all other parameters are held constant. The length of each bar reflects the degree of influence that parameter has on the ICER, with longer bars indicating greater impact. The orange bars represent the ICER when the parameter is at its high value, and the blue bars represent the ICER when the parameter is at its low value. The parameters are listed in descending order of their impact on the ICER, from top to bottom.

**Supplementary table 1: Additional scenario analysis tNGS compared to in-country DST practice**

| **Country** | **Comparator** | **DALYs** | **Cost** | **ICER  ($ per DALY averted, 95% uncertainty ranges)** |
| --- | --- | --- | --- | --- |
| **Decreasing probability of death among those without treatment** | | | | |
| South Africa | XpertXDR+pDST | 0.51 | $3,223 | Ref. |
|  | tNGS | 0.50 | $3,401 | $ 18,592 |
| Georgia | XpertXDR+pDST | 0.50 | $3,035 | Ref. |
|  | tNGS | 0.49 | $3,180 | $25,371 |
| India | LPA and pDST | 0.56 | $981 | Ref. |
|  | tNGS | 0.50 | $971 | Dominates LPA and pDST |
